# Therapeutic efficacy of Pudilan Xiaoyan Oral Liquid (PDL) for COVID-19 in vitro and in vivo

**DOI:** 10.1038/s41392-020-0176-0

**Published:** 2020-05-08

**Authors:** Wei Deng, Yanfeng Xu, Qi Kong, Jing Xue, Pin Yu, Jiangning Liu, Qi Lv, Fengdi Li, Qiang Wei, Linlin Bao

**Affiliations:** NHC Key Laboratory of Human Disease Comparative Medicine, Beijing Key Laboratory for Animal Models of Emerging and Remerging Infectious Diseases, Institute of Laboratory Animal Science, Chinese Academy of Medical Sciences and Comparative Medicine Center, Peking Union Medical College, Beijing, China

**Keywords:** Infectious diseases, Drug regulation

**Dear Editor**,

Coronavirus disease 2019 (COVID-19) caused by severe acute respiratory syndrome coronavirus-2 (SARS-CoV-2), has rapidly swept through the worldwide, with more than 3 million confirmed cases. Until now, no vaccine or effective therapeutic measures are provided to prevent the SARS-CoV-2 infection. Existing medicines have some strong advantages on pharmacokinetics, known side effects, safety and dosing regimens.^1^ Although remdesivir and chloroquine could effectively inhibit the replication of SARS-CoV-2 in vitro,^2^ no medicine candidates have been evaluated in vivo by using animal models with SARS-CoV-2 infection.

Pudilan Xiaoyan Oral Liquid (PDL) is a traditional Chinese medicine preparation composed of Indigowoad Root (*Isatis Indigotica*), Bunge Corydalis (*Corydalis Bungeana*), Mongolian Dandelion (*Taraxacum Mongolicum*), Scutellaria Amoena (*Scutellaria Baicalensis*). PDL has the functions of clearing away heat and detoxification, cooling blood and removing blood stasis, *etc*. It also has strong antiviral and antibacterial effects, and has been widely used in treatment of mumps, pharyngitis, children’s acute tonsillitis, acute bronchitis, and other respiratory diseases.^3^ Herein, we evaluated the efficiency of the PDL against SARS-CoV-2 via in vitro and in vivo studies, which provide the insight for the treatment of the COVID-19.

We initially compared viral replication in PDL-treated or PBS-treated Vero E6 cells with SARS-CoV-2 infection. As shown in Fig. 1a, PDL with a half-maximal effective concentration (EC_50_) = 1.078 mg/mL, half-cytotoxic concentration (CC_50_) = 8.914 mg/mL, selectivity index = 8.27 could effectively suppress the viral replication, implying that virus infection was potently blocked by PDL. SARS-CoV-2-infected hACE2 transgenic mice were treated with PDL to show the potential efficacy.^4^ Twelve SARS-CoV-2-infected hACE2 mice were randomly assigned to the two groups, PDL-treated group and model control group. PDL-treated mice were administrated by intragastric route with PDL (4 mL/kg) from 1 h post virus inoculation, then once daily for 5 days. A notable increase of body weight was observed in PDL-treated mice at 1 day post infection (dpi, *p* = 0.0120), 3 dpi (*p* = 0.0020), and 5 dpi (*p* = 0.0006) compared to the weight loss of model control group after SARS-CoV-2 infection (Fig. 1b). Next, the viral RNA copies of lung were significantly reduced in SARS-CoV-2-infected hACE2 mice with PDL treatment compared with model control group at 3 dpi (****p < 0.0001, *t* = 27.94, df = 4) and 5 dpi (*p* = 0.0021) (Fig. 1c). These data indicated that PDL had a potent inhibitory effect against SARS-CoV-2 in vitro and in vivo, as well as improved the weight loss caused by the viral replication.

**Fig. 1:**
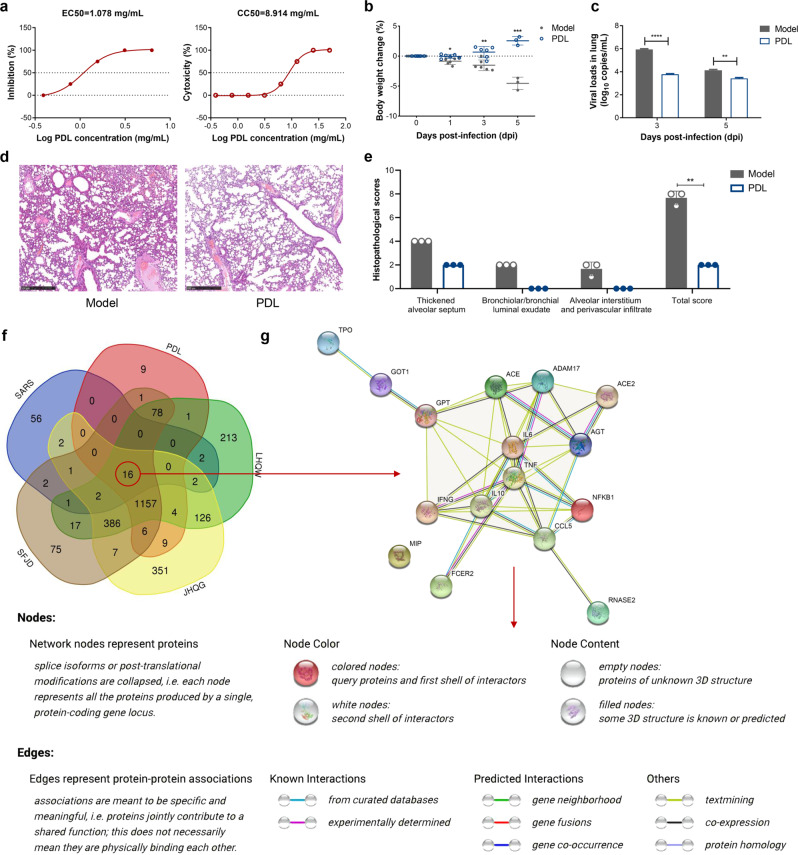
Therapeutic efficacy of PDL against SARS-CoV-2 in vitro and in vivo. **a** Cytotoxic effect and antiviral activity of PDL against SARS-CoV-2 in Vero E6 cells. EC_50_ of PDL for inhibiting SARS-CoV-2 infection was determined by the reduction of CPE. **b** Weight loss in SARS-CoV-2-infected hACE2 transgenic mice with or without PDL treatment. **c** Changes of viral loads from lung tissues detected by qRT-PCR at 3 dpi and 5 dpi. **d** Histopathological analyses of lung tissues in SARS-CoV-2-infected hACE2 transgenic mice with or without PDL treatment. (Bar = 500 μm). **e** Histopathological scores of PDL-treated group and untreated control group, including the lung samples at 5 dpi. The scoring index based on severity of lung histopathology was shown in Supplementary information, Table S3. **f** Venn diagram of SARS, PDL, JHQG, LHQW, and SFJD targeted genes. **g** An association network of PDL-targeted proteins associated with SARS. Their size is proportional to the enrichment measure provided by STRING. All the experiments were performed in triplicates and data represent the mean ± SEM (*p < 0.05, **p < 0.01, ***p < 0.001, ****p < 0.0001)

To evaluate the efficacy of PDL against pneumonia caused by SARS-CoV-2 infection, the histopathological changes were observed in PDL-treated mice and control mice. In SARS-CoV-2-infected hACE2 mice, lung tissues at 5 dpi had diffuse moderate pneumonia with interstitial hyperplasia. Moreover, the alveolar interstitium was thickened with inflammatory cells, and inflammatory cells infiltration around blood vessels were found. In contrast, the lung tissues from PDL-treated mice showed the mild interstitial pneumonia along with small amounts of inflammatory cells infiltrated, albeit the alveolar interstitium expanded (Fig. 1d). The degree of pulmonary inflammation in the two groups was determined and compared semi-quantitatively, and the results showed that the inflammation in PDL-treated mice was significantly reduced compared with that in the untreated control mice (*p* = 0.0034, Fig. 1e). These data indicated that the pneumonia in SARS-CoV-2-infected hACE2 mice was relieved after PDL treatment.

The PDL contains four herbs composed of 181 ingredients. According to disease enrichment analyses of PDL ingredients targeted genes in therapeutic target database (TTD), PDL could have the potential effect on the asthma (*p* = 2.41E−03), chronic obstructive pulmonary disease (*p* = 2.45E−03) which are closely relative to COVID-19 (Supplementary information, Table S1). Since JinHuaQingGan Granules (JHQG), LianHuaQingWen Capsules (LHQW), and ShuFengJieDu Capsules (SFJD) had been recommended for the treatment of SARS-CoV-2 infected patients in the guideline of novel coronavirus pneumonia treatment (V7), 84 SARS-associated genes were mined by the DisGeNET and 16 co-targeting genes (ACE, IL10, ACE2, IL6, ADAM17, GOT1, MIP, AGT, NFKB1, CCL5, RNase2, FcεR2, TNF, GPT, TPO, IFN-γ) in SARS, PDL, JHQG, LHQW, and SFJD were analyzed by Venn diagram (Fig. 1f). For STRING analysis, protein–protein interaction enrichment between PDL and SARS indicated the physical contacts of high specificity established among these 16 proteins (Fig. 1g, *p* = 3.82E−14), and the effective ingredients in PDL compounds related to these 16 proteins were analyzed in Supplementary information, Table S2. As reported, PDL could attenuate pathological damage of lung and reduce the pro-inflammation cytokines in sera such as IL-10 and TNF-α through the protein-metabolite network.^5^ In this study, IL-10 and TNF-α were included in 16 targeted proteins. However, whether these proteins are involved in immune-mediated anti-inflammation and anti-viral processes required further experimental data.

Therefore, our results combined with the analyses of bioinformatics and network pharmacology indicated that PDL exhibited potent anti-SARS-CoV-2 activity and better outcome in vitro and in vivo, which may be clinically used for the treatment of pneumonia caused by SARS-CoV-2 infection alone or cocktailed with other effective antivirals.

## Data Availability

All raw data are available from the corresponding author on reasonable request.

## References

[1] Li, G. & Clercq, E. D. Therapeutic options for the 2019 novel coronavirus (2019-nCoV). *Nat. Rev. Drug Discov.*10.1038/d41573-41020-00016-41570 (2020). 10.1038/d41573-020-00016-0 32127666

[2] Wang, M. et al. Remdesivir and chloroquine effectively inhibit the recently emerged novel coronavirus (2019-nCoV) in vitro. *Cell Res*. 10.1038/s41422-41020-40282-41420 (2020). 10.1038/s41422-020-0282-0 PMC7054408 32020029

[3] Feng L (2018). Pudilan xiaoyan oral liquid alleviates LPS-induced respiratory injury through decreasing nitroxidative stress and blocking TLR4 activation along with NF-ΚB phosphorylation in mice. J. Ethnopharmacol..

[4] Bao, L. et al. The pathogenicity of SARS-CoV-2 in hACE2 transgenic mice. https://www.biorxiv.org/content/10.1101/2020.02.07.939389v1 (2020). 10.1038/s41586-020-2312-y 32380511

[5] Tian G (2020). GC-MS based metabolomic profiling of lung tissue couple with network pharmacology revealed the possible protection mechanism of Pudilan Xiaoyan Oral Liquid in LPS-induced lung injury of mice. Biomed. Pharmacother..

